# Tyrosinase enzyme purification and immobilization from *Pseudomonas* sp. *EG22* using cellulose coated magnetic nanoparticles: characterization and application in melanin production

**DOI:** 10.1007/s11274-023-03796-w

**Published:** 2023-11-10

**Authors:** Salim Mohamed Abd El-Aziz, Ahmed Hassan Ibrahim Faraag, Ayman Meselhi Ibrahim, Ashraf Albrakati, Marwa Reda Bakkar

**Affiliations:** 1 Chemistry Department, Faculty of Science, Helwan University, Cairo, Egypt; 2 Botany and Microbiology Department, Faculty of Science, Helwan University, Cairo, Egypt; 3 Physics Department, Faculty of Science, Helwan University, Cairo, Egypt; 4 Department of Human Anatomy, College of Medicine, Taif University, Taif, Saudi Arabia; 5 School of Biotechnology, Badr University in Cairo, Badr City, Cairo 11829 Egypt

**Keywords:** Apoptosis, Immobilization, Magnetic nanoparticles, Melanin, Tyrosinase

## Abstract

Melanin is a brown-black pigment with significant roles in various biological processes. The tyrosinase enzyme catalyzes the conversion of tyrosine to melanin and has promising uses in the pharmaceutical and biotechnology sectors. This research aims to purify and immobilize the tyrosinase enzyme from *Pseudomonas* sp*.* *EG22* using cellulose-coated magnetic nanoparticles. Various techniques were utilized to examine the synthesized nanoparticles, which exhibited a spherical shape with an average diameter of 12 nm and a negative surface potential of − 55.7 mV with a polydispersity index (PDI) of 0.260. Comparing the immobilized magnetic tyrosinase enzyme with the free enzyme, the study’s findings showed that the immobilized tyrosinase enzyme had optimal activity at a pH of 6 and a temperature of 35 °C, and its activity increased as the concentration of tyrosine increased. The study investigated the antibacterial and anticancer bioactivity of the enzyme’s melanin product and found that it exhibited potential antibacterial activity against a multi-drug resistant strain including *S. aureus and E. coli*. The produced melanin also demonstrated the potential to decrease cell survival and induce apoptosis in initiation cells.

**Supplementary Information:**

The online version contains supplementary material available at 10.1007/s11274-023-03796-w.

## Introduction

Nanoparticles, due to their small size and large surface area, have had a significant impact on many aspects of human life. This has led to rapid growth in the field of nanotechnology as a prominent area of research (Marathe and Doshi 2015).

Scientists have expressed interest in nanoparticles, which are particles that are smaller than 100 nm in size (Wu et al. 2008). Nanoparticles are whether organic or inorganic (Liu et al. 2020). Nanoparticles are considered complex molecules because of their three-layer structure. The nanoparticle’s core layer is the central part, while the surface layer can be modified with various molecules or metal ions. Additionally, the outermost layer has different chemical properties compared to the core (Shin et al. 2016).

The physical structure of substances determines their dimensionality, allowing them to exist in zero dimensions (0D), one dimension (1D), two dimensions (2D), or three dimensions (3D) (Zhou et al. 2021; Asimakoula et al. 2022; Yavaşer et al. 2023).

Nanoparticles, which are materials with at least one dimension that is less than 100 nm, have been extensively researched for their ability to deliver drugs to specific tissues and improve their resistance to enzyme degradation (Du et al. 2018; Wahane et al. 2021; Fang et al. 2023). The classification of nanoparticles involves various criteria, including their size, shape, surface properties, and material composition, which can affect their interaction with biological systems and their therapeutic efficacy (Georgelin et al. 2010; Yiu and Keane 2012; Khan et al. 2017; Jia et al. 2022). Understanding these properties is crucial for designing and optimizing nanoparticles for biomedical applications.

Polysaccharides derived from cellulose, a plant-based material obtained from sources such as cotton, corn cobs, wood, bagasse, and stalks, are an abundant and renewable resource on our planet (Jabareen et al. 2021). This versatile polymer presents significant biotechnological potential and has gained popularity as a raw material in recent years. The exceptional mechanical properties of nanocellulose in particular have garnered growing interest in scientific research over the past decade (Jin et al. 2017). The elongated chain structure of cellulose has been found to be useful in creating a wide range of nanoparticle varieties and adsorbents. Additionally, the use of cellulose has been demonstrated to provide effective stabilization of nanoparticles. These findings are supported by studies such as those conducted by Xu et al. (2019).

Magnetic nanoparticles have been widely researched as a platform for immobilizing enzymes (Varghese et al. 2021; Osuna et al. 2023). Enzyme immobilization involves attaching enzymes to a support material, such as nanoparticles, to improve their stability, reusability, and catalytic efficiency (Pragya et al. 2021; Zhu et al. 2021; Maftoon et al. 2023). Magnetic nanoparticles offer several advantages as enzyme immobilization platforms, including their large surface area, high surface-to-volume ratio, and magnetic properties, which enable easy separation and recycling of the enzyme-nanoparticle complex from the reaction mixture (Vaghari et al. 2016; Xue et al. 2022). Furthermore, magnetic nanoparticles can be functionalized with various surface chemistries to enhance enzyme loading, stability, and specificity. Several methods have been employed to immobilize enzymes on magnetic nanoparticles, such as covalent binding, adsorption, encapsulation, and cross-linking. The resulting enzyme-nanoparticle conjugates have been shown to have improved catalytic properties, such as higher reaction rates, selectivity, and stability, making them attractive for various biotechnological applications, including biosensors, biocatalysis, and bioremediation (Varghese et al. 2021; Zhao et al. 2022; Osuna et al. 2023).

The capping of magnetic nanoparticles is crucial for their various applications, such as catalysis, drug delivery, and environmental cleanup, as it allows for precise manipulation of micro-sized objects at the microscopic scale (Lu et al. 2007; Faraji et al. 2010). Kaur et al. (2023) have developed a method of immobilizing cellulases on chitosan-functionalized magnetic nanoparticles (Ch-MNCs) for use in biomass hydrolysis, Tyrosinase was immobilized on carboxyl functionalized silica-coated magnetic nanoparticles for the first time to be used for fishing of tyrosinase’s ligands present in complex plant extract (Zhao et al. 2022).

Tyrosinase is an enzyme containing copper that can catalyze the o-hydroxylation of monophenols to catechols, which is also known as monophenolase or cresolase activity. Its substrate, tyrosine, can be converted into an o-diphenol called 3,4-dihydroxyphenylalanine (DOPA) by tyrosinase. The same enzyme can then further oxidise DOPA to produce an o-quinone known as dopaquinone (Zaidi et al. 2014; Biundo et al. 2020).

The production of melanin pigments relies on a complex series of enzymatic and non-enzymatic reactions that transform the o-quinone intermediate into melanin pigments (Slominski and Paus 1993; Ito and Wakamatsu 2003; Hearing 2005).

Tyrosinases are enzymes that can be found in various organisms, including plants, fungi, actinomycetes, and bacteria, both eukaryotic and prokaryotic. The *tyr2* tyrosinase gene was identified by analyzing the genome of *Trichoderma reesei.* The gene encodes a protein that possesses a signal sequence and can be overexpressed in its native host with the aid of a potent promoter (Selinheimo et al. 2006).

Extracting the tyrosinase enzyme involves its acquisition from the culture supernatant. Although obtaining tyrosinase from certain sources can be challenging due to limited availability and purity, isolating bacterial tyrosinase is comparatively simple (Faccio et al. 2012; Mayowa and Marilize 2022).

*Pycnoporus cinnabarinus* and *P. sanguineus* are two fungi species that have been reported to produce high levels of tyrosinase. However, the production of tyrosinase in these fungi can be affected by various factors, including culture conditions and strain variability. Therefore, quantitative assays in submerged cultures have been employed to investigate the production of tyrosinase in these strains and to optimize the culture conditions for maximum enzyme production (Varghese et al. 2021; Osuna et al. 2023)*.*

The tyrosinase gene in *Escherichia coli* was induced using a bacteriophage T7 promoter, leading to the production of melanin pigments in agar plates and liquid cultures upon the addition of copper and tyrosine (Zaidi et al. 2014).

The primary goal of this research is to assess the potential of magnetic nanoparticles for purifying and immobilizing the tyrosinase enzyme using tyrosine as a ligand. Additionally, we aim to study the efficacy of the immobilized tyrosinase enzyme in generating melanin and explore its potential as a biocatalyst for melanin production. Furthermore, we intend to investigate the biological properties of synthesized melanin, particularly its antibacterial and anticancer characteristics. By achieving these objectives, we hope to contribute to the advancement of magnetic nanoparticle and tyrosinase enzyme applications in biotechnology and medicine.

## Materials and methods

### Chemicals

The microbiological media used in this study were procured from HiMedia Laboratories Pvt. Ltd (India) and prepared according to the manufacturer's instructions before each experiment. Dehydrated media were reconstituted as per the provided guidelines. Tryptone soy medium (TSB and TSA), Muller-Hinton agar (MHA), and Kings medium A base were utilized in this study. Analytical grade chemicals and solvents were sourced from Sigma-Aldrich or other specified suppliers. The liquid compounds used for cell culture were obtained from a local supplier (El-Ezaby Pharmacy Inc, Egypt) and stored at 4 °C. To promote in vitro cell growth and proliferation, it was crucial to employ a nutrient-rich medium containing balanced salts and essential compounds, such as Roswell Park Memorial Institute (RPMI-1640) medium.

### Cell line and culture conditions

The HepG2 ATCC® HB-8065™ human cancer cell line was obtained from the Tissue Culture Department at VACSERA in Dokki, Egypt. The cells were cultured in RPMI-1640 medium, which was supplemented with 10% heat-inactivated fetal bovine serum (FBS), 1% antibiotic-gentamicin solution, and 1% sodium pyruvate. To maintain optimal growth conditions, the cells were kept at a temperature of 37 °C with a 5% carbon dioxide and 95% humidity atmosphere. They displayed an epithelial morphology and adhered to the culture flask. The cells were grown until they reached 80% confluence in 75 cm^2^ flasks before being harvested, counted, and washed with phosphate-buffered saline (PBS). All materials and equipment used in the study were procured from Lonza, Belgium.

### Bacterial isolate

The Bacteriology lab at the Faculty of Science, Helwan University graciously provided a bacterial isolate that exhibited a distinct green dispersed pigment. This isolate, designated as EG22, was selected as a pure colony in a previous study and maintained on TSA slants for further examination. It was sub-cultured on a monthly basis and stored at a temperature of 4 °C to support its growth.

### Sequencing and phylogenetic analysis of 16S rRNA

#### DNA extraction and PCR

Genomic DNA from the bacterial strain was isolated using the NucleoSpin® DNA RapidLyse Kit (Macherey–Nagel) following the manufacturer's protocol. To amplify the 16S rRNA gene, Polymerase Chain Reaction (PCR) was performed on a Bio-Rad T100™ Thermal Cycler, using two universal primers: an upstream primer (5-AGA GTT TGA TCC TGG CTC AG-3) and a downstream primer (5-ACG GCT ACC TTG TTA CGA CTT-3). The PCR reaction mixture was prepared according to the manufacturer's instructions, with 1 µl of genomic DNA and 1 µl of each specified primer. KAPA HiFi HotStart ReadyMix (2x) from KAPA Biosystems (12.5 µl) was added, along with 9.5 µl of RNase-free water, resulting in a total volume of 25 µl. The 16S rRNA gene was amplified through 35 cycles of denaturation at 94 °C for 20 s, annealing at 57 °C for 20 s, and extension at 72 °C for 20 s, with an initial denaturation at 94 °C for 3 min and a final extension at 72 °C for 5 min. The PCR product was purified using the QIAquick PCR Purification Kit (Qiagen) and unidirectionally sequenced using the high-throughput sequencer NovaSeq 6000 (Illumina, San Diego, CA, USA) at BGI Genomics in China. The 16S rRNA gene sequence was analyzed using Geneious Prime 2021 to generate a phylogenetic tree, which facilitated the identification of the bacterial strain and its evolutionary lineage.

### Preparation of magnetic nanoparticles for tyrosinase purification

Magnetic nanoparticles were synthesized following the methods described by Zahedifar et al. (2019) and Ghamari Kargar et al. (2021) with some modification. The synthesis of magnetic Fe3O4 nanoparticles was achieved by treating carboxymethyl cellulose (CMC) with a 30% solution of ammonia hydroxide, resulting in the formation of a quaternary ammonium salt (Huang et al. 2016). This particular salt has strong reduction capabilities, allowing it to reduce metal ions and generate magnetic nanoparticles. A solution of Fe (III) chloride and Fe (II) sulphate was prepared and quickly mixed, followed by stirring for 30 min. 30% ammonium hydroxide was added to the solution to achieve a black color, and then a mixture of 50 ml of 0.1% carboxymethylcellulose solution was added. The resulting magnetic nanoparticles were washed with alcohol and distilled water until pH 7.0 was reached. Next, 1 gm of cellulose coated magnetic nanoparticles (Si-CMC-MNPs) were mixed with 5% tetra ethyl ortho silicate (TEOS) in a solution containing 100 ml ethanol and 50 ml purified water. The resulting hybrid nanoparticles were collected, washed with ethanol and purified water, and then dried under reduced pressure at room temperature.

To synthesize Tyr-Si-CMC-MNPs, 1 g of prepared Si-CMC-MNPs were mixed with an aqueous solution containing 0.4 g/l of tyrosine. The pH of the magnetic nanoparticle suspension was adjusted to 9.0 using a 1 M NaOH solution. The mixture was heated to 90 °C and stirred for 6 h, then cooled to room temperature. The suspension was washed with alcohol and distilled water, and the final volume of the suspension was adjusted to 100 ml using distilled water.

### Characterization of synthesized magnetic nanoparticles

UV–vis spectroscopy was conducted using the UVS-260D spectrophotometer to investigate absorption maxima in the range of 190–800 nm. The Nanotechnology Centre at Helwan University, Egypt, utilized the Zeta Sizer Nano ZS instrument to determine the size and zeta potential of MNPs.

To evaluate the surface charge of the particles, a 100 μl sample of the nanoparticles was combined with 1 ml of deionized water and subjected to a 150-mV electric field at ambient temperature. The size and morphology of the nanoparticles were examined at the Nano Technology Centre in the Egyptian Petroleum Research Institute using JEOL electron microscopy. The examination was conducted at room temperature using a copper grid coated with amorphous carbon. A drop of the solution was placed on the grid and allowed to dry before further investigation. The transmission electron microscope (TEM) was set to 80 kV to observe the grid. The PerkinElmer ATR Sample Base Plate DIAMOND FTIR spectrometer, which is situated at the Central Laboratory of the Faculty of Science in Helwan University, Egypt, was utilized to directly examine the functional groups of the magnetic nanoparticles (MNPs) within the spectral range of 4000–450 cm^−1^ (Sterner et al. 2008).

### Purification of tyrosinase enzymes

200 µl/ml of Tyr-Si-CMC-MNPs were added to a solution containing a lysate of *Pseudomonas *sp*. EG22* after being disrupted using an ultrasonic homogenizer for 2 min at 10% amplitude (Model 150VT Ultrasonic Homogenizer, Biologics, USA), then, tyrosinase enzyme bind to the surface of the Tyr-Si-CMC-MNPs through the interaction between the tyrosine ligand of the nanoparticles and the enzyme. Following a gentle shaking period of 2 h at 25 °C, an external magnetic field is utilized to separate the magnetic nanoparticles with the attached tyrosinase enzyme from the remaining proteins in the solution. Phosphate buffer solution (0.1 M, pH 7.0) is used to wash the magnetic nanoparticles in order to remove any proteins that are bound nonspecifically. Finally, the tyrosinase enzyme is extracted by using an elution buffer containing l-tyrosinamide, which competes with l-tyrosine residues on the surface of the Tyr-Si-CMC-MNPs to release the enzyme that is attached to the nanoparticles. The elution buffer consists of 50 mM Tris–HCl at pH 7.5, 300 mM NaCl, and 250 mM l-tyrosinamide in a final volume of 10 ml.

### SDS–polyacrylamide gels (SDS-PAGE)

The protein samples were prepared by adding a loading buffer containing Tris–HCl (pH 6.8), glycerol, SDS, 2-mercaptoethanol, and bromophenol blue. The resulting mixture was then diluted to a concentration of 15 μg per lane and cooled. The proteins were separated using SDS-PAGE with a separating gel and stacking gels in an electrophoresis unit at 120 V for 2.5 h. After electrophoresis, we stained the gel with Coomassie brilliant blue G-250 for 1 h and then destained it with distilled water (Lawrence and Besir 2009).

### Immobilization of tyrosinase with magnetic nanoparticles

The method of introducing amino groups to CMC magnetic nanoparticles (CMC-MNPs) involves the use of a bifunctional crosslinker known as 1-ethyl-3-(3-dimethylaminopropyl) carbodiimide (EDC) and an amino-containing compound called ethylenediamine (EDA). The procedure begins with the dispersion of 10 mg of CMC-MNPs in 10 ml of buffer solution such as phosphate-buffered saline (PBS) at a pH of approximately 4.5. Subsequently, 10 mg of EDC and 5 mM of a coupling agent hydroxybenzotriazole (HOBt) are added to the solution.

To immobilize tyrosinase, 250 mg of NH2-CMC-MNPs were placed in a 250 ml Erlenmeyer flask containing a 2.0% glutaraldehyde solution in phosphate buffer (0.1 M, pH 7.0). The NH2-CMC-MNPs were activated and washed with 0.1 M acetic acid solution and 0.1 M phosphate buffer. Subsequently, the activated aminated CMC magnetic nanoparticles (NH2-CMC-MNPs) were utilized to immobilize tyrosinase on their surface (Ozalp et al. 2015). After purification, the NH2-CMC-MNPs were mixed with phosphate-buffered saline to achieve a concentration of 10 mg/ml. The resulting diluted MNPs were then combined with 200 µl of the tyrosinase enzyme and incubated with gentle agitation at 25 °C for 3 h (Zhou et al. 2013, 2014). Once immobilized, the tyrosinase product (Tyr@ase-NH_2_-CMC-MNPs) was purified using dialysis and concentrated through magnetic separation.

### Melanin production and extraction

Melanin production involved adding 100 mg of tyrosinase-coated magnetic nanoparticles to a solution with 0.4 g/l of aqueous tyrosine. A 10^–3^ M copper sulphate solution (100 µl) was then added and incubated at room temperature for 30 min. Melanin extraction was performed using the nanoparticles and a suitable pH and temperature for binding. The eluted melanin solution was then centrifuged to remove remaining nanoparticles, and the extracted melanin was obtained as a dried powder using freeze-drying. The yield of extracted melanin was determined by measuring its concentration with a spectrophotometer, calibrated with a standard melanin solution. This process utilized tyrosinase-coated magnetic nanoparticles for melanin binding and elution with a buffered solution for dried powder extraction. The absorbance of melanin at 475 nm was measured according to Osuna et al. (2023).

### Optimization of tyrosinase activity for melanin production

Immobilized and free tyrosinase enzymes were used to investigate melanin production under different conditions. First, a standard curve was established using known concentrations of melanin and their corresponding absorbance values at 470 nm. Then, the absorbance at 470 nm for the immobilized and free enzyme at different pH values ranging from 4 to 8 were created using appropriate chemicals and adjusted with NaOH or HCl, temperatures were measured at 30 °C, 35 °C, and 40 °C, and the effect of tyrosine concentrations at 0.1 M, 0.05 M, and 0.01 M were estimated, and the stability of the immobilized enzyme over extended time intervals were investigated.

### Antibacterial activity of melanin pigment

Antibacterial activity of melanin produced by *Pseudomonas *sp*. EG22* was evaluated using agar paper disc method (Balouiri et al. 2016) against resistant strain of *Citrobacter fruendii*. Briefly, surface of sterilized MHA was spread with 40 µl of adjusted overnight bacterial culture of 5 log CFU/ml. After that, discs of sterilized papers of about 7 mm diameter were prepared and incorporated with 20 µl of partially purified melanin pigment. The plates were incubated at 37 ℃ for 18–24 h and subsequently examined for bacterial growth and the presence or absence of clear zones. Any clear zones that formed were then measured in millimeters.

The zone of inhibition assay was performed to evaluate the susceptibility of four pathogenic bacteria (*E. coli* clinical strain, *E. coli* ATCC 25922, *S. aureus* clinical strain, and *S. aureus* ATCC 25923) against four antibiotics (amikacin, vancomycin, penicillin-sulbactam, and streptomycin) and melanin. The assay was carried out using the Kirby-Bauer disk diffusion methods (Clinical and Laboratory Standards Institute (CLSI) 2018). Briefly, bacterial suspension with 0.5 McFarland turbidity was spread onto Mueller–Hinton agar plates, and antibiotic disks were placed onto the plates. After incubation for 24 h at 37 °C, the zone of inhibition was measured in millimeters using a caliper. The assay was performed in triplicate, and the results were expressed as mean ± standard deviation (SD).

### Assessment of cell cytotoxicity by MTT assay

The HepG2 cell line was first rinsed with PBS and treated with trypsin for detachment. After a 24-h incubation period, the cells were cultured in a 96-multiwell plate at a density of 104 cells per well, using a complete RPMI-1640 growth medium. The cells were then treated with a chemotherapeutic drug. Next, a serial dilution technique was used to introduce distinct cell culture mediums containing varying concentrations of melanin pigment into the wells. This procedure was repeated three times for each well. The cell monolayers were subsequently exposed to melanin and incubated for 48 h at a temperature of 37 °C in a CO_2_ incubator. Following the incubation period, the melanin-containing medium was removed, and the cells were rinsed twice with PBS. Each well was then treated with 0.5 mg/ml of MTT, which is a yellow compound called methyl thiazolyl tetrazolium bromide and incubated at 37 °C for 4 h. Once the medium was discarded, dimethyl sulfoxide (DMSO) was added to dissolve the formazan crystals. Finally, the absorbance of the resulting color was measured at 570 nm using an ELISA reader (Boster Immunoleader, USA) to determine the percentage of viable cells.

To determine the cell survival rate, the absorbance of the treated wells was divided by that of the control wells and then expressed as a percentage. This calculation was performed for each distinct melanin concentration tested. Based on the obtained data, a correlation between the concentration of a specific melanin and cell viability was plotted to generate the survival curve. From this curve, the half-maximal inhibitory concentration (IC50) of the cancer cell line was determined (Buch et al. 2012).

### Detection of apoptosis by flow cytometry

The IC50 concentration of melanin was first determined using the MTT assay. This value was then divided into IC50/2 and IC50/4 concentrations, which were used to treat the cancer cell line in 25 cm^2^ culture flasks with a starting cell density of 1 × 106 cells for 24 h. Following the treatment, the cells were trypsinized, collected by centrifugation, and washed with ice-cold PBS buffer. The resulting cell pellets were then resuspended in 100 μl of binding buffer, which contained Annexin V-FITC and PI staining solution. The cell suspension was incubated in the dark at room temperature for 15 min, and 400 μl of binding buffer was added. The cells were then analyzed using a COULTER® EPICS® XL™ flow cytometer (Beckman Coulter Co., France). The flow cytometer was used to determine the proportion of cells that were undergoing apoptosis or necrosis, based on Annexin V-FITC and PI staining (Moradi 2018; Nowacka et al. 2022; Thanh et al. 2022).

## Results

### 16S rRNA gene sequence analysis

**Fig. 1:**
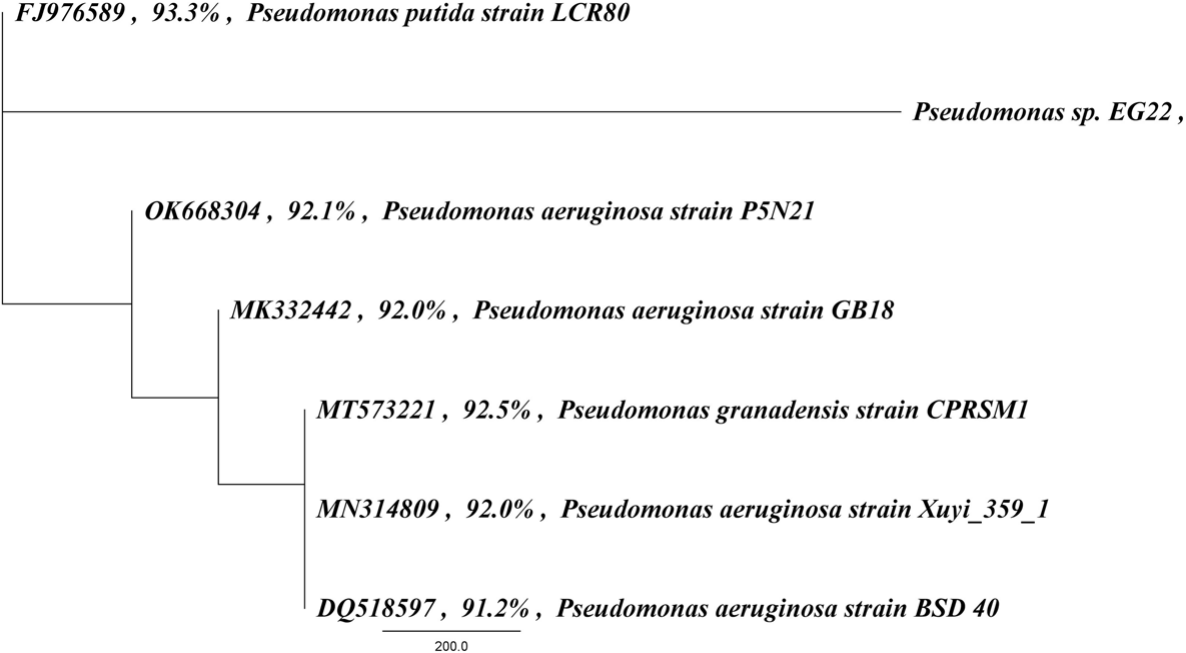
Phylogenetic analysis of *Pseudomonas* sp. *EG22* strain 16S rRNA genes using MUSCLE sequence alignment

The PCR product, which was amplified using primers specific to *Pseudomonas* sp*.* EG22, was sequenced to determine its genetic similarity to other known bacterial strains. The resulting sequence was then analyzed using the BLASTN algorithm and compared to sequences in the GenBank database. The analysis revealed that *Pseudomonas* sp*.* EG22 had a 93.3% similarity with *P. putida* strain LCR80 and a 92.5% similarity with *P. granadensis* strain CPRSM1 (Fig. 1), indicating that it is closely related to these bacterial strains.

### UV–visible spectrophotometer

**Fig. 2:**
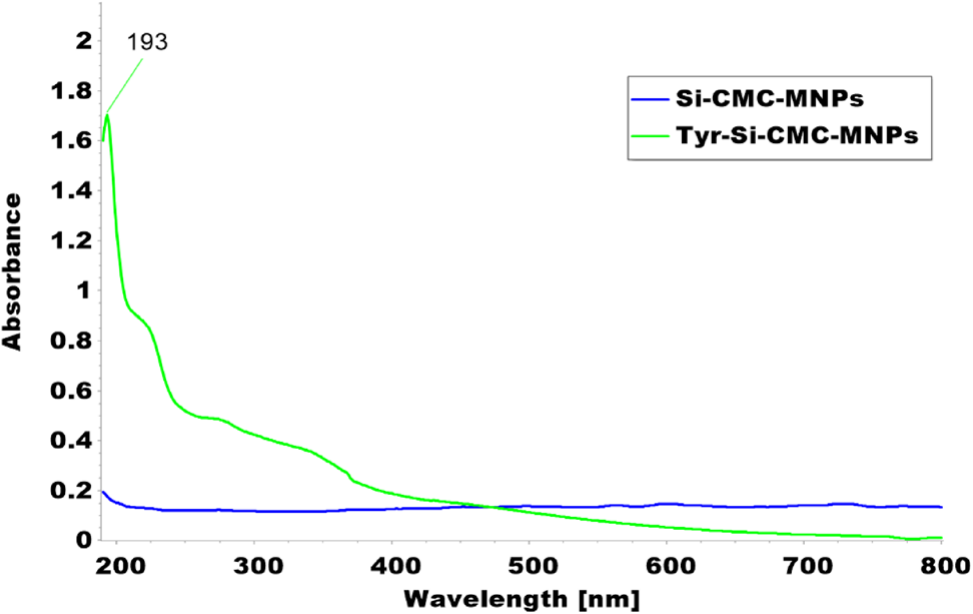
UV–Vis absorption spectra of Tyr-Si-CMC-MNPs and Si-CMC-MNPs

The success of tyrosine conjugation to magnetic nanoparticles coated with CMC and silicate (Tyr-Si-CMC-MNPs) was determined using UV spectrophotometer analysis. The analysis revealed a peak at 193 nm, indicating successful conjugation of tyrosine to the magnetic nanoparticles. In contrast, magnetic nanoparticles coated with CMC and silicate alone (Si-CMC-MNPs) showed no peak at 193 nm, indicating that the peak in the Tyr-Si-CMC-MNPs was specific to the presence of tyrosine and not due to the coating materials. This indicates that the conjugation was specific to the presence of tyrosine Fig. 2.

### Zeta size and surface potential

**Fig. 3:**
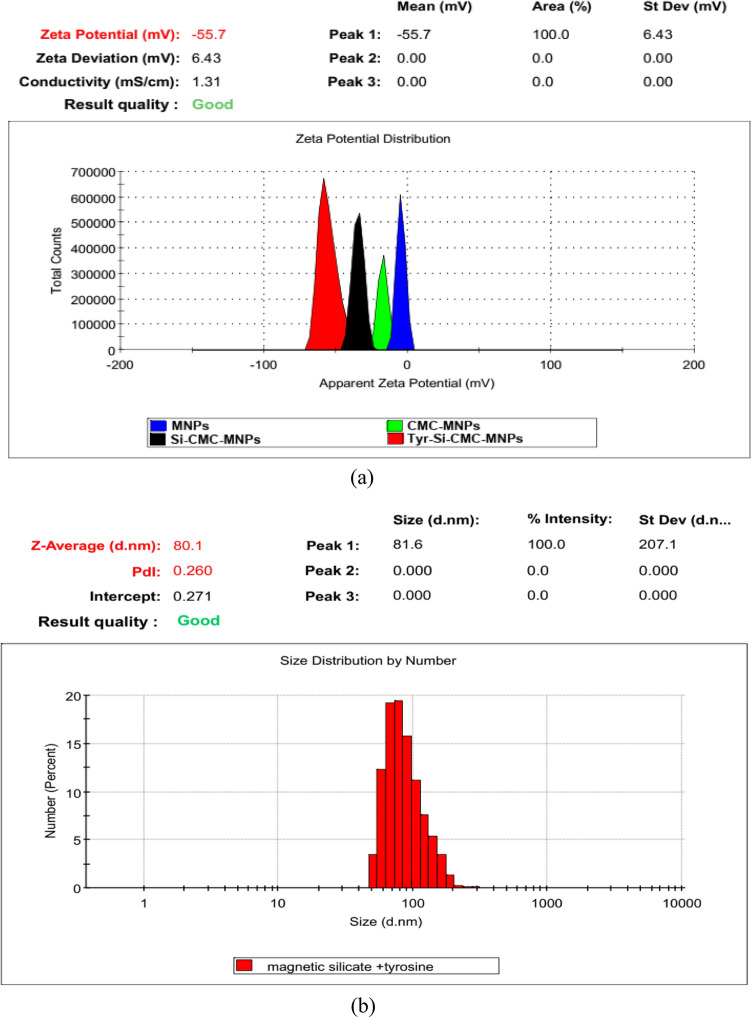
Size and surface potential of magnetic, magnetic silicate, magnetic silicate tyrosine conjugate

The magnetic nanoparticles displayed a surface charge of − 1.94 mv, whereas the magnetic CMC had a potential of − 20.4 mv, the magnetic CMC silicate beads had a potential of − 36.9 mv, and the magnetic silicate tyrosine conjugate had a potential of − 55.2 mv. The average diameter of the CMC silicate tyrosine magnetic nanoparticles was 81.6 nm, with a polydispersity index (PDI) of 0.260. The nanoparticles exhibited a surface potential of − 55.7 mV, indicating their exceptional stability. The surface potential of the magnetic nanoparticles was found to be − 1.94 mv, while the surface potential for magnetic CMC was − 20.4 mv, magnetic CMC silicate beads were − 36.9 mv, and magnetic silicate tyrosine conjugate was − 55.2 mv (Fig. 3a). The average size of the CMC silicate tyrosine-coated magnetic nanoparticles (Tyr-Si-CMC-MNPs) was determined to be 81.6 nm, with a polydispersity index (PDI) of 0.260 (Fig. 3b).

### Transmission electron microscope (TEM)

**Fig. 4:**
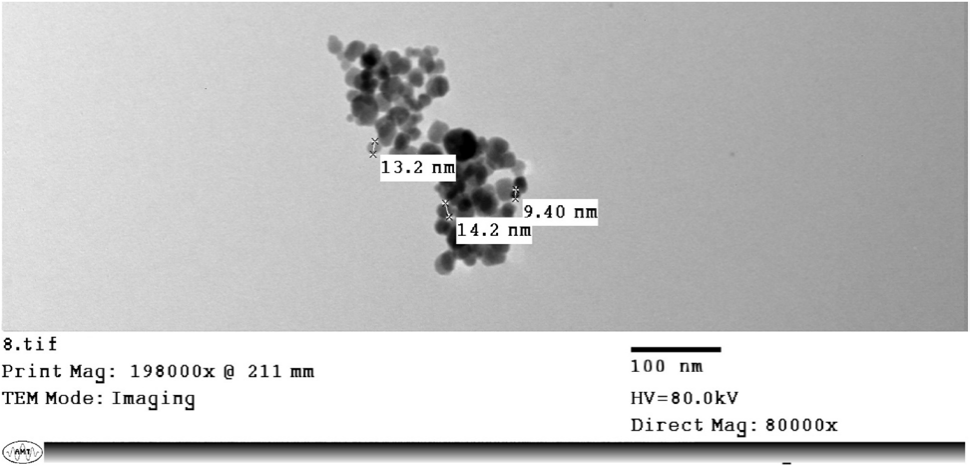
Characterization of size and morphology of synthesized Tyr-Si-CMC-MNPs

TEM imaging was used to characterize the magnetic silicate tyrosine conjugate. Figure 4 show TEM images of the conjugate, revealing its spherical shape. The average particle size of the magnetic nanoparticles, as determined from TEM measurements, was 12.27 nm with a standard deviation of 2.01 nm. The TEM images revealed that the particles were well-dispersed and did not show any aggregation, which suggests that the tyrosine conjugation did not affect the stability of the nanoparticles. This observation further confirms the successful synthesis of the magnetic silicate tyrosine conjugate.

### FT-IR analysis

**Fig. 5:**
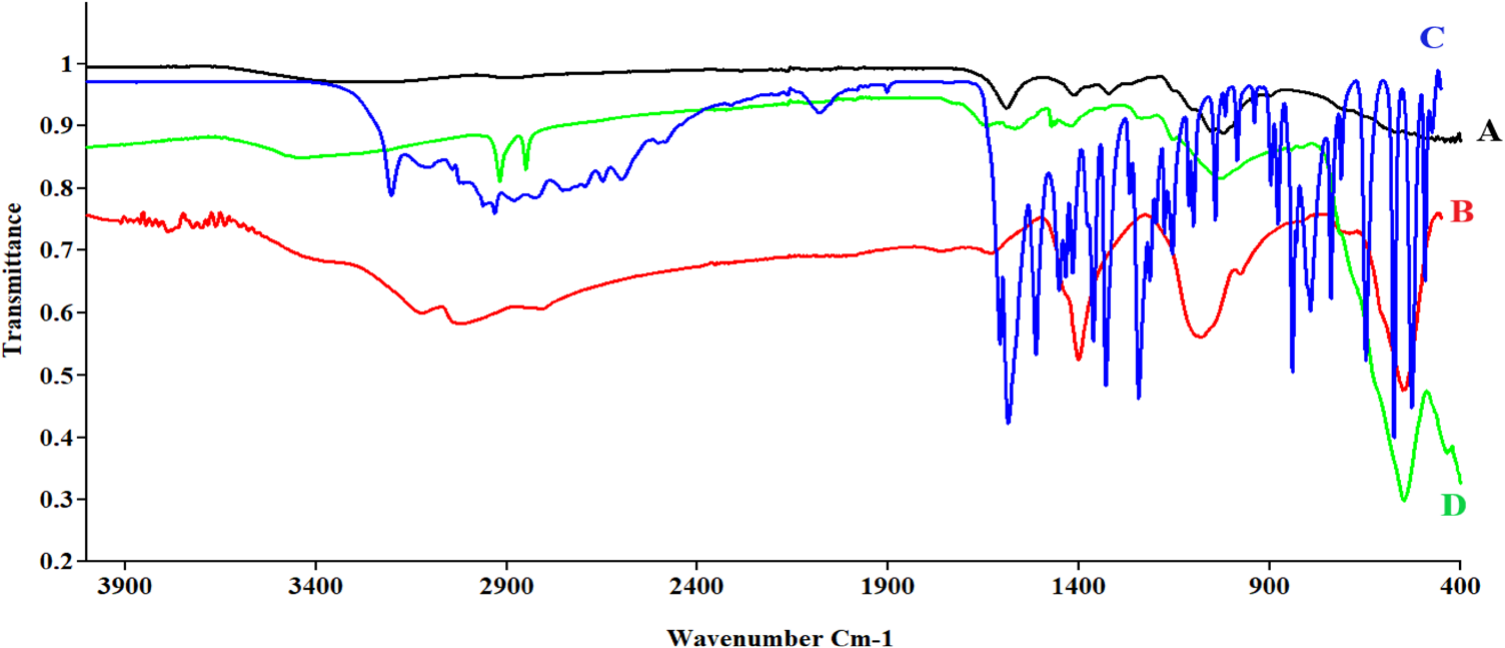
Fourier transform infrared (FTIR) spectroscopic analysis of **A** CMC, **B** MNPs, **C** tyrosine, and **D** Tyr-Si-CMC-MNPs

**Fig. 6:**
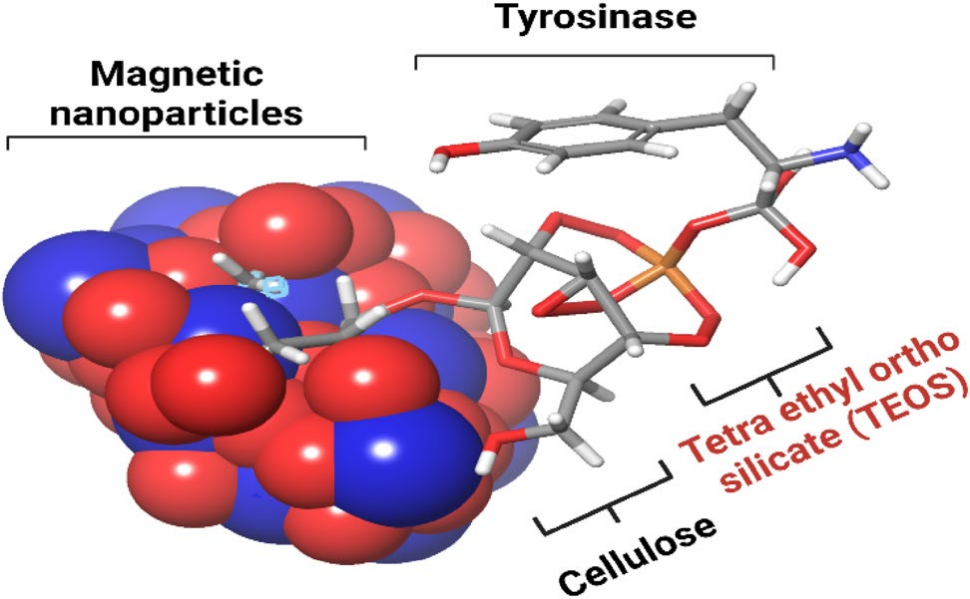
Magnetic nanoparticles coated with carboxy methyl cellulose silicate and tyrosine

Figure 5 presents the FT-IR spectra of cellulose, MNPs, tyrosine, and Tyr-Si-CMC-MNPs to compare the functional groups involved in metal ion reduction and nanoparticle stability. The O–H group stretching mode is ranging from 3650 to 3250 cm^−1^ in all spectra. The absorption bands at around 547 and 551 cm^−1^ in the MNPs and Tyr-Si-CMC-MNPs spectra correspond to the Fe–O stretching mode (Fig. 5), indicating the presence of iron oxides in the samples. This also suggests that the reduction of metal ions during nanoparticle synthesis involves the formation of Fe–O bonds. The FT-IR spectra of tyrosine and Tyr-Si-CMC-MNPs show additional bands at around 1580 cm^−1^, corresponding to the N–H bending vibration of the amide group in tyrosine. This suggests that tyrosine is involved in the functionalization of the nanoparticles. The binding of magnetic carboxy methyl cellulose silicate to tyrosine is further illustrated in Fig. 6, where it can be seen, that tyrosine is attached to the surface of the nanoparticles through a covalent bond with silicate. This binding mechanism is likely to contribute to the stability of the nanoparticles in solution.

### Protein gel electrophoresis

**Fig. 7:**
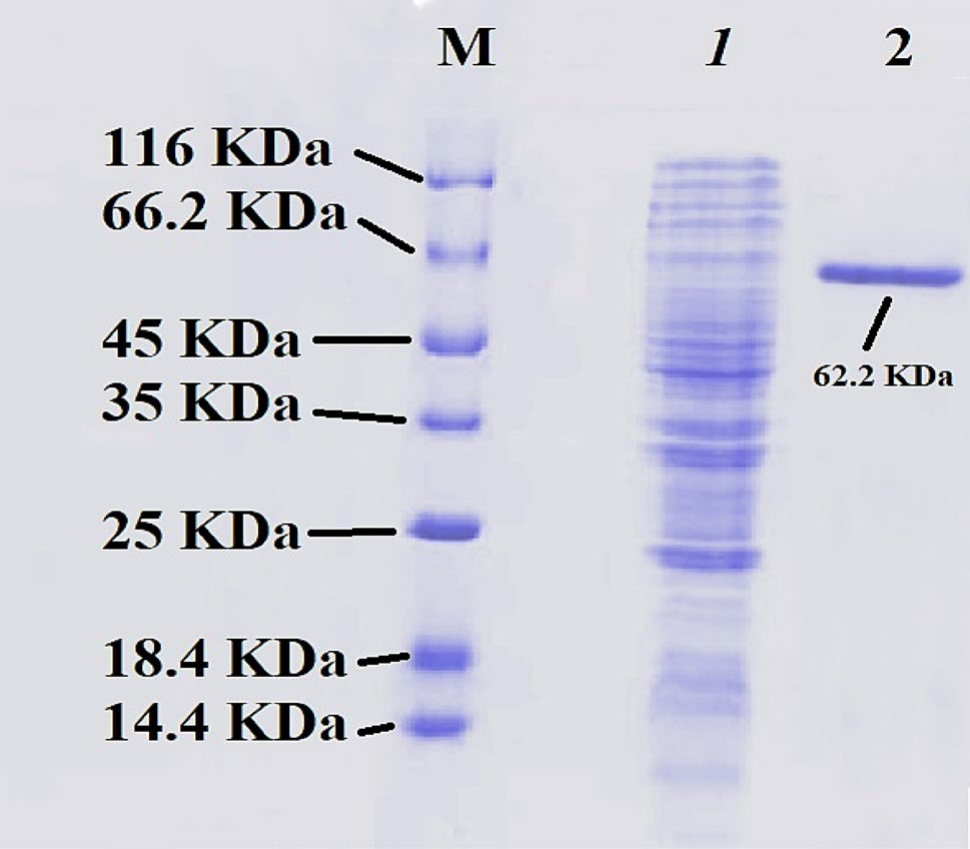
Comparative gel profile analysis of purified enzyme proteins: the protein marker in lane 1, *pseudomonas* sp. EG22 lysate profile in lane 2, and the purified enzyme from magnetic nanoparticles in lane 3

Based on Fig. 7, it can be observed that the protein gel profile of the purified tyrosinase enzyme using magnetic nanoparticles has a molecular weight of approximately 62.2 KDa. This indicates that the electrophoretic purity of the enzyme is high, and that the purification process has been successful in isolating the target protein from other potential contaminants.

### Enzyme activity

**Fig. 8:**
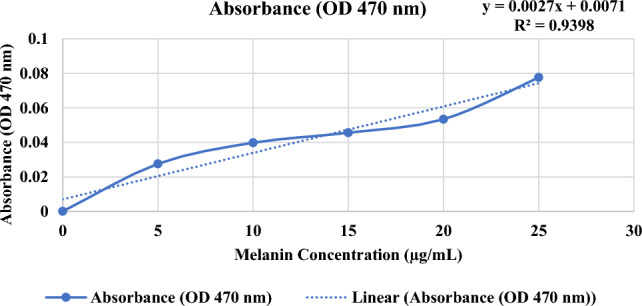
Standard curve for melanin pigment production

The standard curve to estimate the concentration of melanin pigment for each absorbance value was shown in Fig. 8. A standard curve was used to establish a relationship between the concentration of melanin and its corresponding absorbance values at 470 nm. The resulting equation of the line of best fit was y = 0.0027x + 0.0071, where y is the absorbance value at 470 nm and x is the concentration of melanin in µg/mL. Using the equation x = (y − 0.0071)/0.0027, the concentration of melanin pigment for each tested condition was estimated. Using this equation, the concentration of melanin pigment was estimated for the data collected in Tables 1, 2, 3, 4.

**Table 1:** Comparative melanin production by immobilized and free tyrosinase enzyme across a pH range of 4 to 8

PH	OD 475 of immobilized enzyme	Concentration of melanin (µg/mL) of immobilized enzyme	OD 475 of free enzymes	Concentration of melanin (µg/mL) of free enzymes
4	0.444	161.8	0.073	24.4
5	0.712	261.1	0.547	200.0
6	0.757	277.7	0.665	243.7
7	0.644	235.9	0.339	122.9
8	0.461	168.1	0.236	84.8

**Table 2:** Comparative melanin production by immobilized and free enzyme at 30, 35, and 40 °C

Temperature	OD 475 of immobilized enzyme	Concentration of melanin (µg/mL) of immobilized enzyme	OD 475 of free enzymes	Concentration of melanin (µg/mL) of free enzymes
30	0.948	348.5	0.75	275.1
35	1.338	492.9	0.825	302.9
40	1.077	396.3	0.095	32.6

**Table 3:** Investigating melanin production by immobilized tyrosinase at varied tyrosine concentrations

Tyrosine concentrations mM	Immobilized enzyme OD 475 nm	Concentration of melanin (µg/mL) of immobilized enzyme
0.01	0.948	348.5
0.1	1.338	492.9
0.5	1.168	430.0

**Table 4:** Assessing the stability of enzyme activity over extended time intervals (1–6 months)

Time in month	Immobilized enzyme OD 475 nm	Concentration of melanin (µg/mL) of immobilized enzyme
1	1.356	499.6
2	1.345	495.5
3	1.323	487.4
4	1.256	462.6
5	0.994	365.5
6	0.893	328.1

**Fig. 9:**
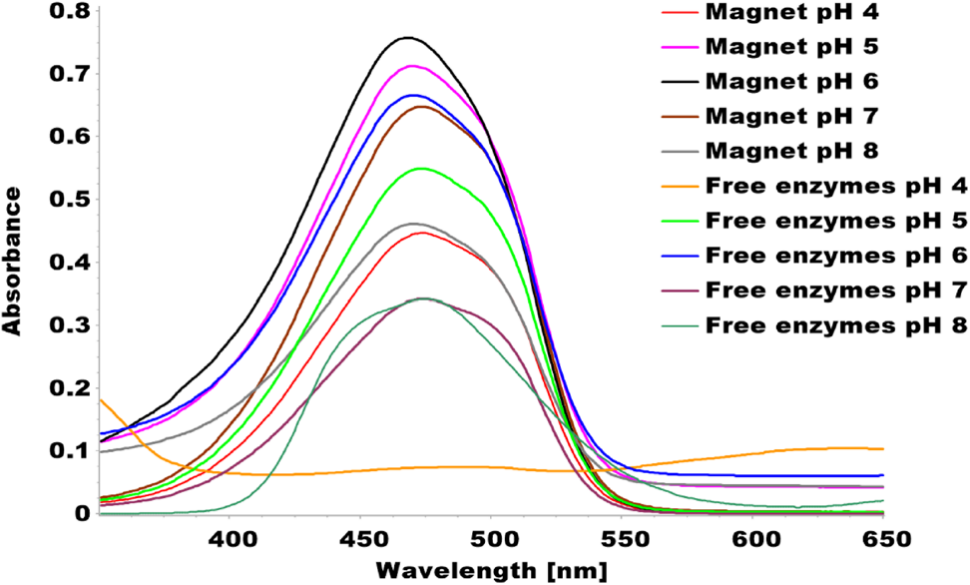
Comparative melanin production of immobilized and free tyrosinase enzymes across varied pH conditions

**Fig. 10:**
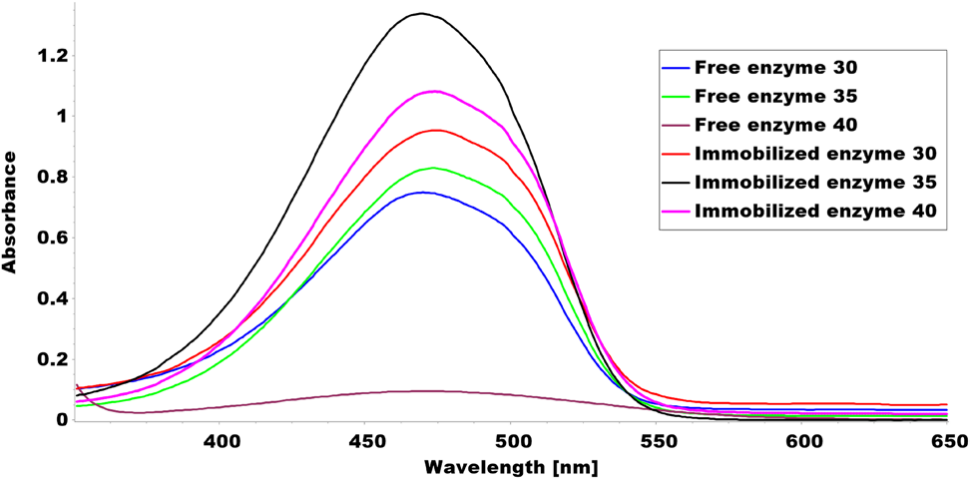
Comparative melanin production by immobilized and free tyrosinase enzyme at varied temperatures

**Fig. 11:**
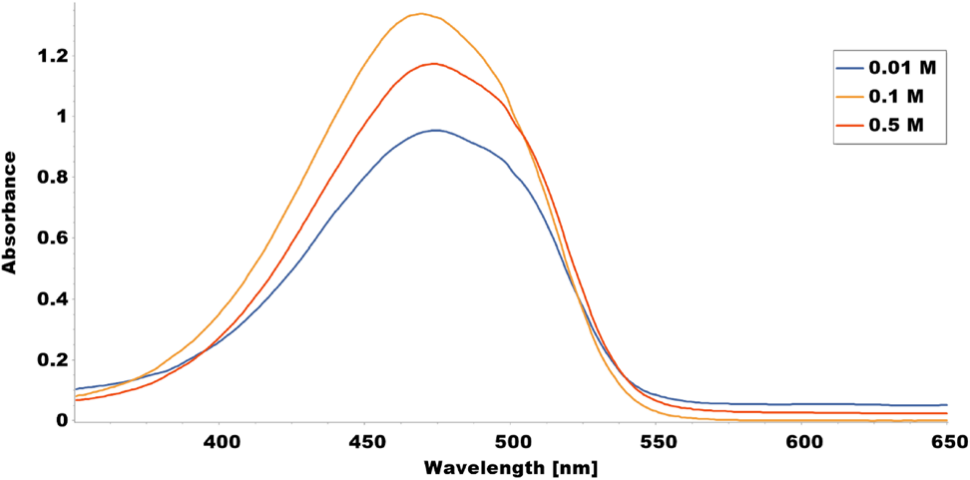
Melanin production by immobilized enzyme at different tyrosine concentrations

**Fig. 12:**
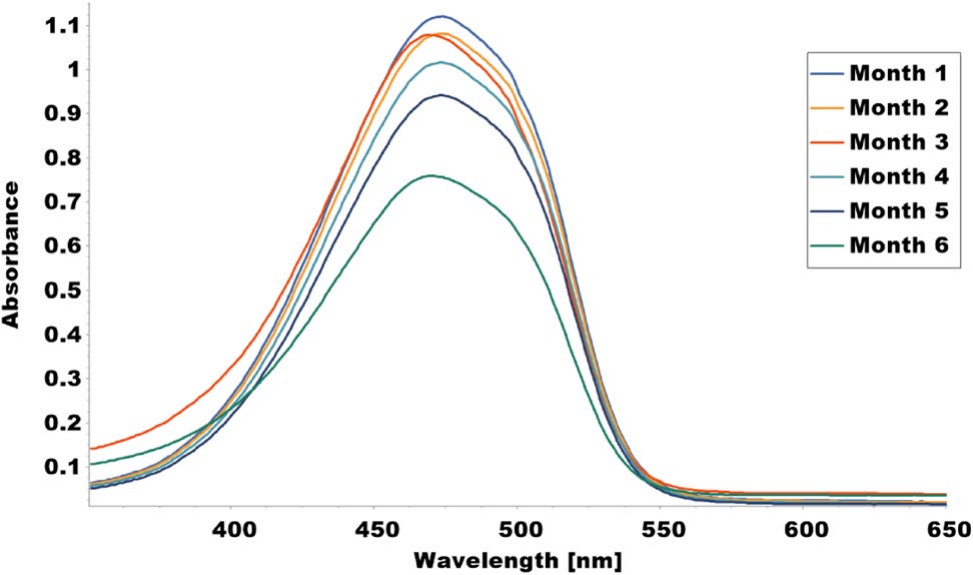
Characterization of tyrosinase enzyme stability in immobilized state during storage

The results showed that immobilized tyrosinase enzyme produced more melanin than free enzyme at all pH values tested (4–8). The highest melanin production was observed at pH 6 for the immobilized enzyme and pH 5 for the free enzyme (Fig. 9 and Table 1). Furthermore, Fig. 10 and Table 2 indicated that both immobilized and free tyrosinase enzymes produced more melanin at higher temperatures (35–40 °C) compared to lower temperatures. However, at 40 °C, the free enzyme produced significantly less melanin than the immobilized enzyme. Increasing tyrosine concentration resulted in higher melanin production for the immobilized enzyme as shown in Table 3 and Fig. 11. Finally, our results showed that the immobilized enzyme retained its activity and melanin production ability for up to 6 months, indicating its potential use in large-scale production of melanin. Finally, the immobilized enzyme retained its activity and melanin production ability for up to 6 months (Table 4 and Fig. 12).

### Antibacterial activity

**Fig. 13:**
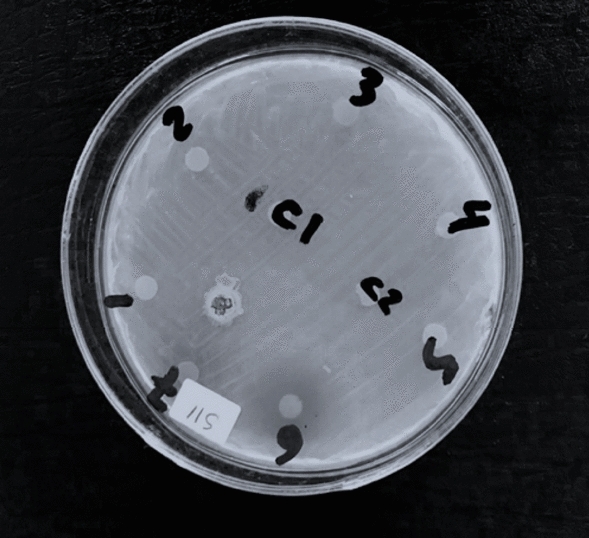
Antibacterial activity of melanin pigment produced by *Pseudomonas* sp. EG22. 1; Gentamycin 10 µg/ml, 2; Norfloxacin 10 µg/ml, 3; Cefixime 5 µg/ml, 4; Cefoperazone 75 µg/ml, 5; Ampicillin 10 µg/ml, 6; melanin 15 µg/ml, C1 and C2; sterile distilled water

**Fig. 14:**
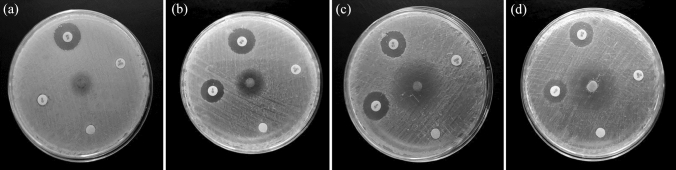
Antibacterial activity of melanin product against; **A** clinical MDR *E*. *coli*, **B** *E*. *coli* ATCC 25922, **C**, clinical *S*. *aureus* and **D**; *S*. *aureus* ATCC 25923

The paper disc method was used to investigate the antibacterial activity of melanin pigment against a multi-drug resistant strain of *Citrobacter fruendii*. Results represented in Fig. 13 showed that the pathogen was sensitive to our melanin product with an inhibition zone of 18 mm, indicating its antibacterial activity. The results of the zone of inhibition assay showed that *E. coli* (clinical strain) and *E. coli* ATCC 25922 were susceptible to amikacin and streptomycin, while no zone of inhibition was observed for vancomycin and penicillin-sulbactam. The *S. aureus* (clinical strain) and *S. aureus* ATCC 25923 were susceptible to vancomycin, and *S. aureus* (clinical strain) was susceptible to streptomycin. No zone of inhibition was observed for other antibiotics in *S. aureus* strains. Melanin showed antibacterial activity against all tested bacteria except *E. coli* (clinical strain), indicating its potential as a natural antibacterial agent Table 5 and Fig. 14.

**Table 5:** Antibacterial activity of melanin product

Pathogenic bacteria	Zone of inhibition in mm ± SD				
	Amikacin	Vancomycin	Penicillin-sulbactam	Streptomycin	Melanin
*E. coli* (clinical strain)	16 ± 0.07	nt	–	–	10 ± 1.0
*E. coli* ATCC 25922	17 ± 0.08	nt	–	13 ± 0.07	15 ± 0.08
*S. aureus* (clinical strain)	nt	20 ± 1.1	–	15 ± 0.0	33 ± 0.0
*S. aureus* ATCC 25923	nt	18 ± 0.09	–	12 ± 0.0	28 ± 1.3

Results are average of three replicates ± standard variation, (nt); not tested, (–); no inhibition zone was detected

### Cell viability assessment

The viability of HepG2 liver cancer cells and the IC50 value of melanin pigment were assessed using the MTT test. Additionally, flow cytometry was employed to identify the various stages of cell death caused by different treatments. These stages included necrotic cells (Annexin-V-/PI+, Q1), late apoptotic cells (Annexin-V+/PI+, Q2), viable cells (Annexin-V-/PI-, Q3), and early apoptotic cells (Annexin-V+/PI-, Q4).

### Melanin response of HepG2 cells

**Fig. 15:**
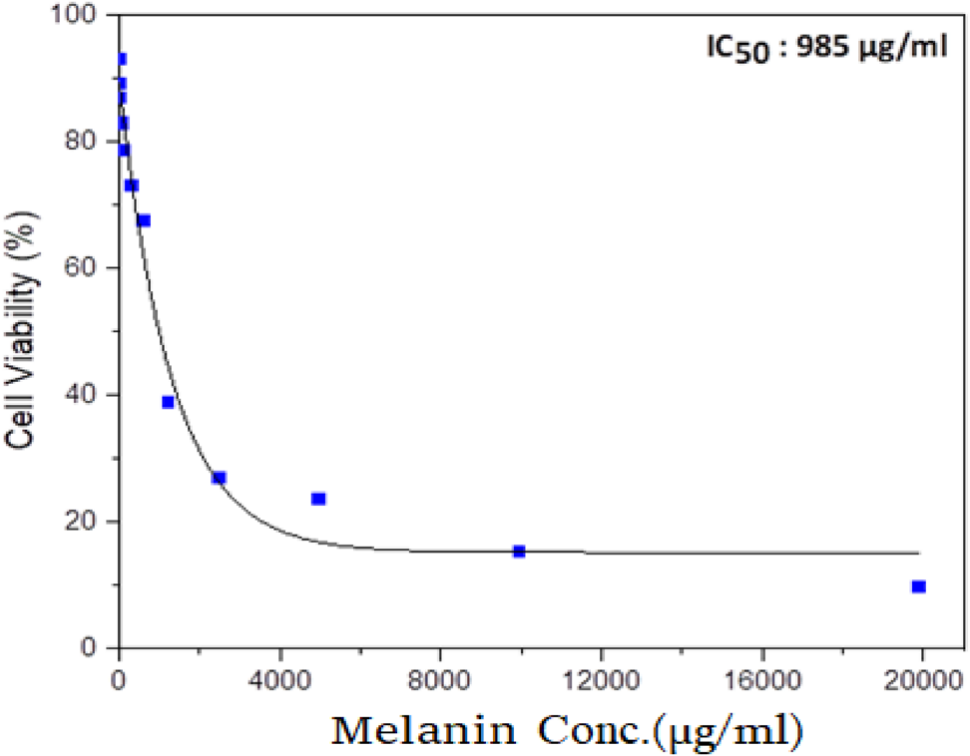
Assessment of cell viability in HepG2 cell line following 48-h in vitro melanin treatment

Figure 15 demonstrates the effect of a melanin IC50 concentration of 985 µg/ml on the HepG2 cancer cell line. The graph displays the average absorbance, expressed as a percentage of the untreated control cells, plotted against various concentrations of melanin.

### Detection of apoptosis in HepG2 cells

**Fig. 16:**
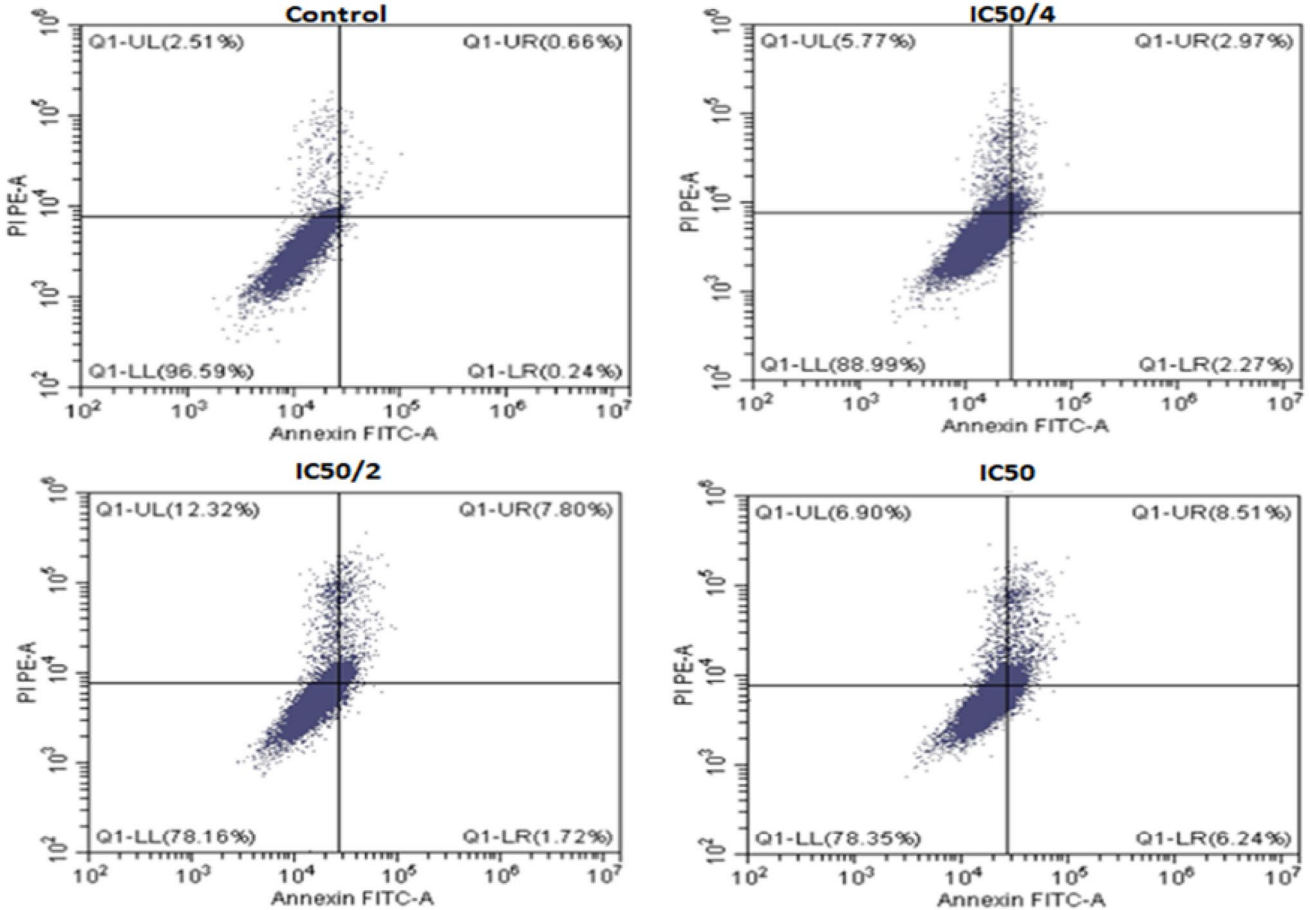
Flow cytometry analysis of melanin pigment effects on HepG2 cell viability

Figure 16 illustrates the different stages of apoptosis induced by melanin pigment in HepG2 cells, as analyzed using Annexin V-FITC/PI staining. The graph shows the percentage of cells in each stage of apoptosis, including viable cells, early apoptotic cells, late apoptotic cells, and necrotic cells, for different concentrations of melanin. The results indicate that the highest percentage of early and late apoptosis (14.75%) was observed when HepG2 cells were exposed to the IC50 concentration of melanin. In contrast, the lowest percentage of early and late apoptosis (5.24%) was observed when cells were exposed to the IC50/4 concentration of melanin. Additionally, untreated cells had a very small percentage of the distinctive apoptotic features.

## Discussion

*Pseudomonas* sp*.* EG22 is also known to produce melanin, and like *Pseudomonas aeruginosa* and *P. putida*, this process relies on the activity of tyrosinase enzymes. The conversion of tyrosine into DOPA and melanin synthesis are key steps in the process of melanin production in these bacteria. Melanin production is an important function of these bacteria and is believed to contribute to their survival and adaptation in various environments (Korner and Pawelek 1982; Jimbow et al. 1993; Kasraee 2002; Ferraz et al. 2021). Tyrosinase enzymes play a crucial role in the synthesis of melanin by converting tyrosine into dihydroxyphenylalanine (DOPA) and subsequently into melanin. Both free and immobilized enzymes can be utilized for melanin production (Rabaey et al. 2005; Das et al. 2013).

Studies have been carried out to purify and immobilize tyrosinase enzymes for various applications, including the synthesis of melanin (Khan et al. 2005; Chomoucka et al. 2010; Zdarta et al. 2020). Different methods have been employed to purify tyrosinase enzymes, including ammonium sulfate precipitation and chromatography using ion-exchange or affinity with specific ligands. Our study aims to purify tyrosinase enzyme using carboxy methyl cellulose coated magnetic nanoparticles and tyrosine as ligand to enhance binding affinity with the enzymes. The purified enzymes have been immobilized on different matrices, including magnetic nanoparticles, to enhance their stability and reusability (Al-Abbasy et al. 2021). Enzymes immobilized on chitosan have been shown to have improved operational stability and reusability compared to free enzymes (Sakono et al. 2019; Verma et al. 2020; Fidalgo et al. 2021). (Liu et al. 2018) developed a technique that combines physical adsorption and covalent crosslinking for immobilization, aiming to overcome the limitations of both noncovalent and covalent coupling methods. In a recent study by Zhao et al. (2022), tyrosinase was successfully immobilized on carboxyl-functionalized silica-coated magnetic nanoparticles. This immobilization technique was used for the first time to extract tyrosinase ligands from complex plant extracts.

Using silica-coated magnetic nanoparticles and immobilizing tyrosinase on them, we achieved successful melanin production. The immobilized enzyme displayed enhanced catalytic activity and improved reusability compared to the unbound enzyme (Abdollahi et al. 2017, 2019). The UV spectrophotometer analysis confirmed the successful binding of tyrosine to the magnetic nanoparticles, as indicated by the presence of a peak at 193 nm. This absorption behavior of tyrosine is consistent with its known characteristics, as it absorbs UV light within the wavelength range of 190–240 nm, attributed to its aromatic ring structure (Chapman 1963; Steyermark 1976; Wong 2015). Based on the peak range observed in this study, it can be inferred that the tyrosine-linked magnetic nanoparticles are composed of tyrosine molecules. The lack of a peak in the UV spectrophotometer analysis of magnetic nanoparticles coated with CMC and silicate alone suggests that the binding process was targeted solely towards tyrosine. This targeted binding is crucial in ensuring that the magnetic nanoparticles are modified with the desired molecule and can be utilized for specific purposes. The incorporation of tyrosine-coated magnetic nanoparticles has shown promise in drug delivery, as it has been observed that the presence of tyrosine improves the cellular uptake of nanoparticles (Chomoucka et al. 2010). The analysis conducted using TEM revealed that the Tyr-Si-CMC-MNPs possessed a spherical shape with an average diameter of 12 nm and provided information on their size and morphology (Murdock et al. 2008).

The spectrum of Tyr-Si-CMC-MNPs shows 3020.25 cm^−1^ –C–H streching, 1401.73 cm^−1^ –C–H bending, the Fe–O–Si stretching vibration at 1025.76 cm^−1^ (Ahangaran et al. 2013; Nalbandian et al. 2015; Lobato et al. 2017; Dawn et al. 2022). The spectrum of tyrosine shows the 3105.72, 3040.60 cm^−1^ =C–H stretching, 2930.03–2825.20 cm^−1^ –C–H stretching, 2080.06 Cl–H stretching, 1606.16 NH_2_ scissoring - 1556, 1584.02 cm^−1^ alkenyl aromatic C=C stretch, 1451 cm^−1^ –CH_2_ bending, 1329.54 cm^−1^. The combination of stretching of C–C–C bonds and the presence of Phenolic OH groups is observed at a wavenumber of 1112.14 cm^−1^. Additionally, the presence of C–N bonds is observed at a wavenumber of 1243.04 cm^−1^, along with the presence of C–O bonds (Anandan et al. 2012). Based on the absorption of IR bands, the cellulose effectively reduced the Fe_3_O_4_NPs and provided a thorough coating of SiO_2_ and tyrosine layers.

The average size of the prepared magnetic nanoparticles, CMC silicate tyrosine (Tyr-Si-CMC-MNPs), was 81.6 nm. These nanoparticles showed a polydispersity index (PDI) of 0.260 and a surface potential of − 55.7 mV, indicating excellent stability (Gutierrez and Flores 2018). At 475 nm, the peak absorbance of dopachrome, a byproduct of the tyrosinase reaction, was measured (Osuna et al. 2023). The study examined the production of melanin by both immobilized and free enzymes at various pH levels from 4 to 8. The findings indicated that melanin production reached its peak at pH 6 for both immobilized and free enzymes. However, the immobilized enzyme consistently generated more melanin than the free enzyme across all pH values.

The enhanced production of melanin by the immobilized enzyme can be attributed to several factors. Firstly, the immobilized enzyme displayed superior stability compared to the free enzyme, resulting in reduced degradation. Secondly, the immobilized enzyme had enhanced access to the substrate, resulting in a higher reaction rate. Finally, the immobilized enzyme was less susceptible to inhibition by inhibitors present in the culture medium (Huang et al. 2012; Brena et al. 2013; Stancu 2020).

The results of this study demonstrate that immobilizing enzymes can enhance the efficiency of melanin production compared to non-immobilized enzymes. This improvement is attributed to the increased stability, improved accessibility to substrate, and reduced susceptibility to inhibition of immobilized enzymes. The findings show that magnetic immobilization of the tyrosinase enzyme increases the concentration of melanin produced, particularly at a pH of 6. This may be due to the enhanced stability of the immobilized enzyme at pH 6, allowing for optimal function and higher melanin production. Furthermore, previous studies have shown that the use of magnetic immobilization can enhance the stability, activity, and reuse of enzymes. This is achieved by providing protection against harsh reaction conditions and reducing the leakage of the enzyme (Imarah et al. 2021; Sharma et al. 2021; Csuka et al. 2022). In a related study, Kaur et al. (2023) developed a method of immobilizing cellulases on chitosan-functionalized magnetic nanoparticles for biomass hydrolysis. This research adds to the growing body of knowledge on the use of magnetic nanoparticles in enzyme immobilization and demonstrates their potential for various applications in biotechnology.

On the other hand, the highest concentration of melanin produced by the free tyrosinase enzyme was observed at pH 6, but it was still lower compared to the melanin produced by the magnetic immobilized tyrosinase enzyme. This difference can be attributed to the instability of the free enzyme at acidic and basic pH levels, which can lead to enzyme denaturation and a decrease in activity (Johnson et al. 2008; Ren et al. 2011; Yu et al. 2012; Sánchez-Ramírez et al. 2014; Vaghari et al. 2016; Khoshnevisan et al. 2017; Li et al. 2017; Bilal et al. 2018; Darwesh et al. 2020; Fauser et al. 2020). Furthermore, the utilization of unbound enzymes frequently leads to decreased enzyme effectiveness because of their limited reusability and vulnerability to deactivation caused by reaction byproducts and other elements. According to Liu et al. (2018), the tyrosinase enzyme that was immobilized using magnetic nanoparticles showed enhanced resistance to changes in pH and temperature, with the greatest resilience observed at pH 7.0 and a temperature of 35 °C.

The observed increase in melanin production with the immobilized enzyme at all three temperatures can be attributed to several factors. Firstly, the enzyme’s stability and activity are enhanced by immobilization, protecting it from denaturation and breakdown and providing an optimal environment for its function. This may explain why the immobilized enzyme produced higher concentrations of melanin compared to the free enzyme at all three temperatures tested. Secondly, the immobilized enzyme's increased accessibility to the substrate could also contribute to the higher melanin production (Khan 2021). The magnetic immobilization technique used in this study may have facilitated contact between the enzyme and substrate, potentially increasing the reaction rate and melanin production. Lastly, the immobilized enzyme was less likely to be inhibited by inhibitors present in the culture medium. This could be because the magnetic immobilization technique allowed for separation of the enzyme from the culture medium, preventing inhibitors from binding to the enzyme and inhibiting its activity (Trindade Ximenes et al. 2021).

The increase in melanin concentration, observed as tyrosine concentration rises, can be attributed to the conversion of tyrosine into melanin facilitated by the tyrosinase enzyme. As the amount of tyrosine increases, there is more substrate available for the enzyme to convert, leading to a higher concentration of melanin. However, once a certain level is reached, the concentration of melanin begins to decline. This decline may be attributed to the inhibition of enzyme activity at high tyrosine concentrations, causing a decrease in melanin production (Wu et al. 2018). The results suggest that the magnetic immobilized tyrosinase enzyme can effectively convert tyrosine to melanin, with the highest concentration obtained at a tyrosine concentration of 0.1. These findings could have potential applications in the development of biosensors or biocatalysts for the detection or degradation of melanin in environmental or medical settings. Further studies could be conducted to optimize the conditions to produce melanin using magnetic immobilized tyrosinase enzyme, as well as to investigate its stability and reusability (Wu et al. 2018; Trindade Ximenes et al. 2021).

The results suggest that the *Citrobacter freundii* strain used in the study displays resistance to all tested antibiotics, even at the prescribed concentrations. The growing concern over the emergence of resistance to multiple antibiotics in healthcare facilities is due to the challenge it presents in treating infections caused by these bacteria. The melanin disc created an inhibition zone of 18 mm, indicating its ability to inhibit the growth of the tested bacterial strain. Melanin, a black pigment produced by *P. aeruginosa*, a Gram-negative bacterium known for causing infections in immunocompromised individuals, has been found to have the potential to suppress the growth of *C. freundii*. Therefore, melanin could be a promising antimicrobial agent. Our melanin product showed potential antibacterial activity against both Gram types of bacteria. This biological activity was previously reported in other studies (Kamarudheen et al. 2019; Deepthi et al. 2021; Jigna et al. 2022; Polapally et al. 2022).

The antibacterial properties of melanin pigment are believed to be due to its ability to bind strongly to the surfaces of bacterial cells. This binding is facilitated by the negative charge of melanin, which allows it to interact with negatively charged molecules on the bacterial cell surface, such as teichoic acids, lipopolysaccharides, and phosphate groups. Once bound, melanin disrupts the cell walls and membranes of the bacteria, leading to the leakage of cellular contents and ultimately causing cell death (Rurián-Henares and Morales 2008). In addition, melanin can interfere with the metabolic pathways of bacterial cells, inhibiting their growth and proliferation. Furthermore, melanin has the ability to generate reactive oxygen species (ROS) when exposed to light, which can induce oxidative damage in bacterial cells and contribute to their demise (Rahmani Eliato et al. 2021).

To assess the anticancer properties of melanin pigment against HepG2 cell lines, the MTT assay was utilized. It is important to note that a 24-h treatment with chemotherapy agents may not significantly reduce cell viability. Therefore, it may be beneficial to evaluate cell viability after 48 h (Yu et al. 2015). Evaluating the effectiveness of a drug at an early stage can provide valuable information, as prolonged exposure may lead to misleading results by suggesting similar effects among different substances. Phosphatidylserine can serve as a specific marker for identifying cells in the early stages of apoptosis. Annexin V-FITC, a compound with a strong affinity for phosphatidylserine, is well-suited for detecting early apoptosis. Additionally, propidium iodide (PI) is a red-fluorescent dye that effectively binds to the nucleic acids in cells, preventing it from entering live cells and early apoptotic cells. However, it does stain late apoptotic and necrotic cells, causing them to exhibit red fluorescence (Sivakumaran et al. 2018)**.**

It was observed in our study that a significant increase in apoptosis was induced when varying concentrations of melanin pigment were administered to HepG2 cancer cells, suggesting a correlation between lower melanin resistance and increased apoptosis. To evaluate the effect of melanin pigment on cell viability, the expression of the apoptotic marker, Annexin V, was measured. Flow cytometry analysis revealed that after 24 h of exposure to melanin pigment, higher levels of total apoptotic cell populations (Q2 + Q4) were exhibited by the HepG2 cells compared to the control cells. This indicates that the effects of melanin pigment were less resisted by the HepG2 cells.

In conclusion, the tyrosinase enzyme from *Pseudomonas* sp*.* EG22 was purified and immobilized using cellulose coated magnetic nanoparticles, and the bioactivity of its melanin product was examined. Results indicated that the optimal pH and temperature for tyrosinase activity aligned with prior reports, and that the immobilized magnetic tyrosinase was considerably influenced by pH and temperature. The study revealed that immobilizing the tyrosinase enzyme on cellulose coated magnetic nanoparticles could be an effective approach for increasing melanin production. Particularly at pH 6, the magnetic immobilization of the enzyme enhanced the concentration of produced melanin, highlighting its potential in melanin production. Furthermore, the results showed that the magnetic immobilized tyrosinase enzyme generated more melanin than free tyrosinase enzyme at all three temperatures. The study also demonstrated the enzyme’s ability to effectively convert tyrosine to melanin, with the highest concentration observed at a tyrosine concentration of 0.1. Overall, the study underscored the potential of immobilized tyrosinase enzyme for enhancing melanin production. The use of melanin pigment exhibited potential in decreasing cell survival and inducing apoptosis in initiation cells. It was observed that HepG2 cells displayed reduced resistance to cytotoxic melanin pigment when treated with the IC_50_ concentration, highlighting the fact that the response of cells to anti-cancer drugs can vary depending on the drug concentration, even under normal conditions.

### Supplementary Information

Below is the link to the electronic supplementary material.

Supplementary file1 (FASTA 1 kb)

Supplementary file2 (TXT 1 kb)

Supplementary file3 (FCS 2739 kb)

Supplementary file4 (FCS 2739 kb)

Supplementary file5 (FCS 2739 kb)

Supplementary file6 (FCS 2739 kb)

## Data Availability

The data supporting the findings of this study, including the raw data files of the flow cytometer (Test1-control.fcs, Test2-quarter IC50.fcs, Test3-half IC50.fcs, and Test4-IC50.fcs), as well as the 16s rRNA sequence and AB1 file for the *Pseudomonas* sp*.* EG22 bacterial strain, have been provided as Supplementary material. Additionally, the analytical methods employed in this research, encompassing the purification and immobilization process of the tyrosinase enzyme using cellulose-coated magnetic nanoparticles, along with the evaluation of its bioactivity in melanin production, are available upon request from the corresponding author, Ahmed H. I. Faraag (email: professor_ahmed85@science.helwan.edu.eg). Due to the sensitive nature of the data and the ongoing research in the field, access to the data will be granted after a reasonable request and with the appropriate confidentiality and ethical considerations.
